# A multicenter randomized controlled trial of aftercare services for severe mental illness: study protocol

**DOI:** 10.1186/1471-244X-13-178

**Published:** 2013-07-01

**Authors:** Ahmad Hajebi, Vandad Sharifi, Mohammad Ghadiri Vasfi, Maziar Moradi-Lakeh, Mehdi Tehranidoost, Masud Yunesian, Homayoun Amini, Arash Rashidian, Seyed Kazem Malakouti, Yasaman Mottaghipour

**Affiliations:** 1 Mental Health Research Centre, Tehran Psychiatric Institute, Iran University of Medical Sciences, Tehran 15745-344, Iran; 2 Department of Psychiatry and Psychiatry and Psychology Research Center, Tehran University of Medical Sciences, Tehran 13337-95914, Iran; 3 Department of Community Medicine, Tehran University of Medical Sciences, Tehran, Iran; 4 Department of Environmental Health Engineering, School of Public Health, Tehran University of Medical Sciences, Tehran, Iran; 5 Department of Health Management and Economics, School of Public Health, Tehran University of Medical Sciences, Tehran, Iran; 6 Psychiatry Department, Imam Hosein Hospital, Shahid Beheshti University of Medical Sciences, Tehran, Iran

## Abstract

**Background:**

Severe mental illness is responsible for a significant proportion of burden of diseases in Iranian population. People with severe mental illnesses are more likely to have high rates of non-attendance at follow-up visits, and lack of an active follow-up system, particularly in the country’s urban areas that has resulted in the revolving door phenomenon of rehospitalizations. Therefore, there is an increasing need for implementation of effective and cost-effective aftercare services.

**Method/Design:**

This is a randomized control trial with the primary hypothesis that aftercare services delivered to patients with severe mental illnesses in outpatient department and patient's home by a community care team would be more effective when compared to treatment as usual (TAU) in reducing length of hospital stay and any psychiatric hospitalization. Patients were recruited from three psychiatric hospitals in Iran. After obtaining informed written consent, they were randomly allocated into aftercare intervention and control (TAU) groups. Aftercare services included treatment follow-up (through either home care or telephone follow-up prompts for outpatient attendance), family psychoeducation, and patient social skills training that were provided by community mental health teams. Patients were followed for 12 months after discharge. The primary outcome measures were length of hospital stay and any hospitalization in the 12 month follow-up. Secondary outcome measures included patients' clinical global impression, global functioning, quality of life, and patient's satisfaction. The trial also allowed an assessment of direct cost-effectiveness of the aftercare services.

**Discussion:**

This paper presents a protocol for an RCT of aftercare services delivered to patients with severe mental illnesses within patients' home or outpatient department. The findings of this study can influence policy and program planning for people with severe mental illnesses in Iran.

**Trial registration:**

IRCT201009052557N2

## Background

### Situation analysis

Studies across the globe report the lifetime prevalence of schizophrenia and bipolar I disorder to be around 1% and 0.72%, respectively [1]. A recent national survey in Iran showed that the lifetime prevalence of psychotic disorders in Iran is 1% [2]. Moreover, it is estimated that there are at least 400,000 patients suffering from severe mental disorders in the country that include schizophrenia, schizoaffective, and bipolar disorder [3].

Improving the services provided for patients with mental disorders is the main priority of mental health care system in the world. Every year up to 30% of the worldwide population suffer from some kind of mental disorders and at least two third of them do not receive any kind of treatment even in developed countries [4]. In the US, 67% of patients and in Europe more than 74% remain untreated [5,6]. Studies have demonstrated that the ratio of patients treated in low and middle income countries is lower than that in the US and the UK. For example in China, 11.1% of cases with severe psychiatric disorders received any treatment during the past 12 months and in Nigeria a minority (10.4%) of patients who sought treatment received adequate therapy [7].

During the past five decades, major strategic changes have been made in the field of providing mental health services in the world [8,9]. In this respect, Iran also tried to improve mental health services and transform it to more efficient community-based services. National integration of mental health services into the primary health care system is considered as the peak of this reform [10]. This program was widely accepted in the rural areas and gained great success. However, implementation of this program in the urban areas was not a success because of inadequacy of the coverage and quality of services [11]. At present, although most psychiatrists and psychologists reside in cities, mental health services in residential areas are inadequate and insufficient, so public-private partnerships are weak and mental health services are not well-regulated [12]. Services are actually limited to psychiatric hospitals and private offices, and community-based out-patient services are rare. Psychiatric hospital beds in large cities are usually 100% occupied and active follow up services do not exist. Discontinuation of treatment, repeated recurrences and relapses and re-hospitalization of patients is a common scenario. As a result, majority of centers face the revolving door phenomenon, which does not help the existing problem of psychiatric bed shortage [13].

Providing active services and follow-up after discharge can have a significant impact on decreasing the relapse and re-hospitalization and improving patients’ clinical condition [14-16]. It can also reduce burden of psychiatric disorders and increase the efficacy of interventions [17]. It is well established that utilization of outpatient aftercare services following episodes of acute inpatient treatment results in better outcomes. Given the scale of the problem and the lack of currently available active aftercare to provide continuous care services for patients with severe mental illness in Iran, an expert group was formed at the Ministry of Health and Medical Education. Based on the evidences and their experiences, they designed a program for providing aftercare services that has three main components; treatment follow-up (through either home care or telephone follow-up prompts for outpatient attendance), family psychoeducation, and social skills training. The evidence for each component of the aftercare service is reviewed in the next section. This study aims to evidence of the effectiveness of this model of aftercare services.

### Literature review

#### Home care

There are different models of home care services but in all of them one or more health care professional(s) offer health care services to patients at the convenience of their home (and not health centers). The most popular of these models are the Assertive Community Treatment (ACT) [16,18-20] and Community Mental Health Teams (CMHT) [21,22]. Other models are also available which are designed for acute and chronic phases of disorders as well as post discharge care. Studies have demonstrated that visiting patients at home and related follow-ups can significantly decrease number of relapses, shorten the hospitalization period [23,24], improve social and occupational functioning [25], and decrease the cost of treatment [14].

#### Telephone prompts

The rate of non-attendance at all types of outpatient clinics are different and is based on setting and specialty [26,27]. Studies demonstrated the national rate for non-attendance at psychiatric clinics is twice that of most other specialties in UK [28]. Non-attendance by patients with psychiatric illnesses in outpatient clinics has an important impact on clinical and economic outcomes [29]. Many factors contribute to this finding including stigma, lack of insight, inappropriate referral and lack of social stability [30].

There are numerous methods which tackle this problem and engage the patient such as telephone prompting [31], with or without specific visits to the home [32], financial incentives [33] and issuing a copy of the referral letter to the appointee [34] and text-based prompts [35]. Follow up by phone includes all the methods employed to encourage patients to show up in clinics and health centers for their follow-up. Patients are usually reminded of their follow-up appointment by phone or mail which is done by a nurse, a social worker, or a physician. A Cochrane’s systematic review showed that by using this method there is a greater chance for the patient to show up for his/her appointment [35].

#### Family psychoeducation

Various studies have shown that different methods of family intervention with the aim of connecting with patients' family and educating and supporting them are effective in decreasing the rate of relapse, hospitalization period and also stress and burden imposed on the patients' family [36]. Previously, the general belief would consider the family responsible for patient’s condition, but at present, this belief has changed and physicians involve the family in the process of treatment and ask for their cooperation based on their abilities and requirements [37,38]. However, providing services for the families as a routine approach has not yet been established [39]. Various clinical guidelines have suggested offering services to patients’ families as the main part of treatment for patients suffering from serious mental disorders [40].

Evidence indicates that patients' family education decrease rate of relapse, risk of re-hospitalization and hospital stay and increased compliance [36,39]. Family intervention can also improve patients’ functioning [41]. In several studies, patients’ families have reported their major needs to be gaining knowledge and learning necessary skills [42,43]. Family psychoeducation could both be as multiple family group formats in outpatient services and as single family (in home or clinic). Group education for families of patients with schizophrenia can change their families’ attitude towards the illness. It can also enhance the knowledge of their family members [44].

#### Social skills training

Since the development of methods for social skills training in 1960s and 1970s, the effect of such trainings on patients who suffer from severe mental disorders, has been widely studied. For evaluating the effectiveness of social skills training during the first 2 decades, the emphasis was on individual studies [45]. In an assessment of previous studies similar results were obtained which are as follow [46-50]: 1) participants learn new social skills; 2) participants can keep the learned skills after the completion of the course; 3) participants can apply the learned skills to real life situations; 4) social skills training improves patients’ functioning and enhances the quality of their social interactions; and 5) social skills training can affect the symptoms, relapse and re-hospitalization to some extent.

Social skills training result in decreasing dependency in daily living activities and related distress. It also has a significant effect on long term prognosis of patients with severe conditions [51]. Such trainings along with group therapy improve patient’s functioning and quality of inter-personal interactions [52]. In a study by Tsung and Pearson (2001) it was revealed that social skills training with regular monthly follow ups can have a significant role in achieving occupational goals [53]. Study of the effects of social skills training in the middle aged and the elderly patients showed their increased ability in performing daily living activities and improving the negative signs [54]. The results of a study revealed social skills training is effective for improving the social skills and self-esteem of patients with chronic schizophrenia [55].

### Objectives

This study aims at evaluating and comparing the clinical effectiveness and direct cost-effectiveness of aftercare services in patients with severe mental disorders with that of usual treatments. The primary objectives are to compare 1) of length of hospital and 2,) readmission rates between intervention (aftercare) and control (TAU) groups in a 12 months follow-up of patients with severe mental illnesses discharged from psychiatric hospitals. The secondary objectives were to compare the following measures between aftercare and treatment as usual groups in a 12 months follow-up of patients with severe mental illnesses discharged from psychiatric hospitals: 1) symptoms severity, 2) global functioning, 3) quality of life, and 4) service satisfaction. The other purpose of the study was to determine the cost-effectiveness of aftercare services vs. treatment as usual (TAU).

We hypothesize that providing services for patients with severe mental disorders (schizophrenia, schizoaffective, and bipolar I disorder) according to the designed aftercare model can decrease the length of hospital stay and hospitalization rate, improve the quality of life and global functioning, increase patient satisfaction, and also be cost-effective when are being compared with routine conventional care.

## Method/Design

### Study design

This is a study of the randomized parallel group controlled trial of effectiveness of the aftercare services that also permits an evaluation of cost-effectiveness of the aftercare services. There is equal allocation of participants between groups.

### Trial inclusion and exclusion criteria

Patients suffering from schizophrenia, schizoaffective or bipolar disorders, who were in the age range of 15 to 65 years, were candidate for this study. These patients were recruited from three psychiatric centers in Tehran at the time of discharge from the hospital and they should live in the catchment area of the hospitals. Another inclusion criterion was that patients have to be living with a family member because this increases the possibility of future follow ups and cooperation with regard to home care. We made sure that our subjects are not suffering from severe physical or neurologic conditions or mental retardation at the same time. Subject inclusion was done by research coordinator in each center. A written informed consent was obtained from both patients and their families.

### Setting

This trial was conducted for patients at three university-affiliated hospitals in Iran (Roozbeh and Iran Hospitals in Tehran and Nour hospital in Isfahan). These are tertiary referral centers that accept patients from all over the country; however most of them are coming from Tehran. These centers provide high quality conventional care services however no outreach, community-based or rehabilitative services are routinely provided.

### Intervention group

The aftercare services included the following three components: treatment follow-up (through either home care or telephone follow-up prompts for outpatient attendance), family psychoeducation, and social skills training.

#### A) Treatment follow-up

The aftercare service consisted of services for two groups of patients: 1) Patients who had severe mental illness with poor compliance that required more assertive follow-up; for example patients with poor medication compliance or those who missed appointments, patients with a very severe and debilitating illness, or patients who were home-bound; this group received home care. Home visits were made once in a month with the exception of the first three months, which were twice per month. Extra visits were made more frequently for unstable patients. In each visit a team of general practitioner and another professional (a social worker or a psychologist) made visits, prescribed medications and provided education and support to patients. 2) Patients with more compliance who needed follow-up but not as assertive as the former group; this group received telephone follow-up prompts to attend the outpatient clinic for the follow-up visits that was usually on a monthly basis. In each outpatient visit a psychiatrist assessed the patients‘clinical status and prescribed medications. Both of these two follow-up modes were different components of the active intervention and the patients inside the intervention group could cross between these two modes of care based on their improvement or deterioration during the study period. All these assignments were made by clinical judgment of supervising psychiatrist of the research team at each center.

Programs for family psychoeducation and social skills training (below) were incorporated into the above-mentioned modes of treatment follow-ups.

#### B) Program for family psychoeducation

This program aims to provide services for families of patients with severe mental disorders. It included three stages: stage 1) The development of initial rapport with families, stage 2): Increasing information and the coping skills of families, including six weekly two-hour multiple family group sessions (each group consists of 6 to 8 families) for telephone follow-up group and six weekly two-hour single-family sessions for home care group, and stage 3) providing support for families through the availability of crisis services, contact with local family organizations, and the formation of self-help groups. The program was based on a culturally adapted and tested manual in Iran [56].

#### C) Program for social skills training

The major rehabilitative component of the service included social skills training that was started after the period allocated to family psychoeducation (usually after 3 months of inclusion in the study). It was provided for the telephone follow-up group at the outpatient settings and included 10 sessions on a weekly basis. But, rehabilitation services were presented for the home visit group on a monthly basis for 9 months at the patient's homes by the home visit team. In the latter group, rehabilitation was mainly based on education of Activity Daily Living and consultation. The aim of the social skills training was to scale up psychosocial functioning, decrease ratings of psychopathology, and also improve level of quality of life. The main frame of the intervention which has been adopted in social skills training was behavioral. It included psychoeducation, modeling, shaping and reinforcement.

### Control group

The control group (TAU) received the same assessments as the intervention group, but they did not receive the aftercare services. They received routine psychiatric clinical care, which included a first visit two weeks after discharge, and once in a month by a psychiatrist or psychiatric resident.

### Trial recruitment process

**Figure 1:**
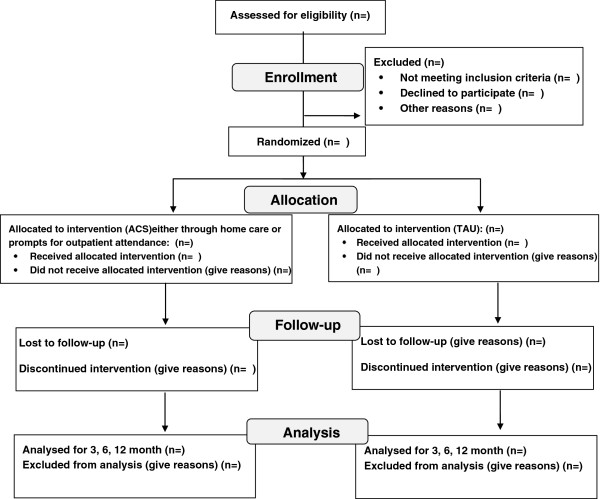
Flow of participants during the conduct of the trial.

Patient selection was performed by a research coordinator who had the responsibility of screening hospital wards 2 times a week. In each time, he screened all patients admitted in the past few days for eligibility criteria. If patients met all of the inclusion and none of the exclusion criteria, they were selected for the study. Afterwards, he contacted subjects to obtain consent. The flow of participants during the conduct of the trial is shown in Figure 1.

### Trial consent procedure

Patients who met the inclusion criteria were informed with verbal and written information regarding the study and then both patients and their guardians were asked to give a written informed consent if they agreed to participate in this study. This was done by the research coordinator at each center. Consent procedures had been designed in a non technical language. It was matched to the literacy levels of the participant and caregiver and was designed in a manner to increase the intake of information. For those patients who meet all inclusion criteria, the research coordinator established a meeting with the patients and their families. In the meeting he/she explained the trial and the purposes of the trial and tried to response to all their questions. Then he asked the patients and the caregivers whether they were interested in participating in the trial. If both were interested to participate, he took written consent. If not, they excluded from the trial and received treatment as usual. The consent procedures and interventions have been approved by the Ethical Committee in Tehran University of Medical Sciences. Subjects had the right to quit whenever they like it. Those who did not consent to participate in the study received the routine and conventional care.

### Trial randomization procedure

After obtaining a written consent, patients were divided randomly into two groups of intervention and control (TAU). There was equal allocation of participants between arms of the study (allocation ratio 1:1). Randomization was provided by an independent statistician at the medical university. Eligible patients were assigned to intervention or control (TAU) groups by stratified balanced block randomization method with allocation concealment. There was a psychiatrist at each center responsible for concealment procedures. After assignment, each patient of Intervention group was classified according to the severity criterion by the research coordinator (see above) and then received the needed mode of care: either home care or telephone prompts for outpatient attendance. Therefore, the latter was not random but based on patients’ needs.

However, to ensure comparability of intervention and control groups with regard to severity of patients’ illnesses we did a stratified randomization procedure. Patients were first categorized according to its severity that included measures for frequent admissions and/or treatment compliance. Then patients in each group of severity (high or low) were randomized into aftercare or treatment-as-usual groups. For example, patients who had an illness with high severity that required home care (in the intervention group) were evenly randomized into two groups.

### Data collection/Management process

The primary outcome measures were the length of hospital stay and any psychiatric rehospitalization during the 12 months post discharge. The secondary outcome measures included: 1) symptom severity, 2) clinical global impression of the illness, 3) global functioning, 4) quality of life, and 5) patients’ satisfaction with service. We also performed a direct cost-effectiveness analysis.

Data were collected from the intervention and control groups at time of index admission, discharge and at each follow-up point. Follow-up ratings in Iran and Nour hospitals were scheduled 3, 6, and 12 months and in another one (in Roozbeh hospital) at 4, 8 and 12 months following discharge and were performed in the outpatients department or at home by an experienced and trained clinician.

We used the following tools in baseline assessment, when the patients were discharging from the hospital, and the follow-up ratings: the Positive and Negative Symptoms Scale (PANSS) for psychotic symptoms [57], the Young Mania Rating Scale (YMRS) for manic symptoms [58], the Hamilton Depression Rating Scale (HDRS) for depressive symptoms [59], the Clinical Global Impression (CGI) for illness severity [60], the Global Assessment of Functioning (GAF) for global functioning [61], the Farsi translation World Health Organization Quality of Life (WHOQOL-BREF) for quality of life [62] and Client Satisfaction Questionnaire (CSQ-8) for the assessment of patients' satisfaction with the services [63].

Data with regard to rehospitalizations and length of hospital stay were carefully collected and evaluated during follow-up assessments by a questionnaire which was designed by the research team and asks questions from patients and their relatives; medical records were reviewed as well. It should be mentioned that in the follow-up ratings a Cost Analysis Questionnaire were also used for calculating direct costs for the 12 month follow-up period. Medical costs included costs of hospitalization, outpatient and community visits, family psychoeducation, social skills training, medicine, and complementary-alternative therapies in both groups and also all payments for providing specific healthcare of the interventions. Non-medical costs included costs of transportation of patients, caregivers and healthcare teams (in the intervention group). Raters were clinical psychologists or general practitioners who were trained in a 2-day workshop on how to use data collection tools before the initiation of the study. Inter-rater reliability of ratings was also examined. We chose raters not from care providers to minimize bias.

Studies show that the rate of “loss to follow up” for ratings in these setting is high [28]. For this reason, patients and their family members were asked to provide their address and phone number at the beginning of the study. One week prior to the follow-up rating appointment, the patient or other named contact were contacted to determine if they would attend their appointment. The day before their appointment, patients were contacted to remind them that their follow-up appointment is due the next day. If the patients were unable to be contacted, or the patients did not show up for their appointment, the follow up team contacted them again. Eventually the research coordinator visited the patients at home and found out if they still stay in that address and also if they want to continue participating in the study. If the patient was no longer living in that address or he/she did not want to participate in the study any longer, he/she was excluded from the study.

### Blinding

The nature of such services prevents adequate blinding of participants. It was not possible to blind the raters who will perform follow-up evaluations on cases and controls because he has a direct contact with patients when rating patients.

### Quality assurance

A single team trained all the care providers and raters in different sites with a single training module. A single guideline was used by all care providers. There were supervisors for each center who oversaw the whole process of the study. They used the same protocol for supervision that consisted of weekly meetings, checking the data collection procedures, supervision of training sessions, etc. The same supervision procedure was employed for the whole process of case recruitment and data collection.

### Sample size calculation

Sample size calculation was based on the length of hospital stay as the primary outcome measure. The following formula was used for the calculation:

$$n=2{\left[z_{\left(1-\alpha /2\right)}+z_{\left(1-\beta \right)}\right]}^{2}\times \left(SD^{2}\right)/d^{2}$$

where d = (delta [difference]). Here delta equals to our estimate of the difference between length of hospital stay in experimental and control groups. In a study on the efficacy of home care service [64], it was shown that the mean hospitalization days in the home care group was about a third of that in the treatment as usual group (14.5 vs. 41.7 days). Also in an unpublished study (Vandad Sharifi, Personal Communication), we found that mean hospital stay of patients with severe mental disorders equals to 45 days with the SD of 18 days. For the present study we aimed for a more conservative and still important difference. We hypothesized that the home care program leads to at least 10 days reduction in hospital stay (×1- × 2 = 10, SD = 18, Cohen's d = 0.6). Sample size was estimated 45 per group; assuming about 30% losses to follow-up, sample size was calculated 60 per group (in total: 120). In one of the centers (Roozbeh hospital) the sample size was 80 per group (in total: 160), because the caseload was higher that allowed recruiting more patients in the same period.

### Planned analysis

Descriptive summaries of socio-demographic and clinical data will be provided for all subjects at different time points. These include means and standard deviations, or proportions. The clinical ratings will be summarized in terms of the total score, and the proportion of patients improving from baseline. An “intention to treat” approach will be employed. Baseline qualitative variables will be compared between the intervention and control group by chi2 test. The quantitative variables will be compared using *t*-test and Mann–Whitney *U* test. The quantitative primary and secondary outcomes measures will be compared using general linear model (GLM) repeated measure analysis. Length of stay will be assessed in each period of follow-up; we analyze it both as the cumulative hospital stay (using *t* test) and as the stay in each follow-up period. The second analysis will be performed by repeated measures analysis of variance analysis.

We calculate all direct medical and non-medical costs of care from a societal point of view. To do that, total costs of care (not just out-of-pocket payments) were considered. Medical costs included costs of hospitalization, outpatient and community visits, family psychoeducation, social skills training, medicine, and complementary-alternative therapies in both groups and also all payments for providing specific healthcare of the interventions. Non-medical costs included costs of transportation of patients, caregivers and healthcare teams (in the intervention group). We divide incremental direct costs (Costs in the intervention group minus control group) by incremental effects (based on primary and secondary outcomes) to calculate incremental cost-effectiveness ratio (ICER). Statistical package for social sciences (SPSS version 18.0) will be used for data analysis.

### Ethical considerations

Before the conduct of the study, patients and their family members were thoroughly informed regarding the services that were provided for them. Then, written informed consent was obtained from the patients and their guardians. The obtained data were strictly confidential. It was well explained to patients that they have the right to quit participating in the study any time they want and if so, they can continue receiving routine conventional therapy offered by the hospital (out-patient or in-patient). In addition, patients were hospitalized whenever it was necessary. If treatment modalities like electroconvulsive therapy were necessary, patient underwent the required treatment. No cost was imposed on patients and all the expenses like the cost of the home visit (but not the cost of medications or hospitalization) were provided from the study budget. No patient group was disadvantaged as clinical care in these patients varies.

Questionnaires were filled out anonymously and were specified only by a code. Using that code, data were entered the database software. Patients’ medical records were maintained in a locked up cabinet in a room in the mental health clinic.

Moreover, in each center there was an external supervisor assigned from Vice Chancellor of Research who oversaw the whole procedure of research and he received progress reports of the project in each center and if necessary he had to make decisions with regard to undesired events.

The study was approved at Tehran University of Medical Sciences Ethics Committee ref: 130-6-2441, and is fully compliant with the Helsinki declaration 2008. The trial is registered with Iranian Registry for Clinical Trials and the allocated unique ID number is IRCT201009052557N2.

## Discussion

This paper presents a protocol for an RCT of aftercare services delivered to patients with severe mental illnesses by community mental health teams within patients' home or at the outpatient clinic. Also, the trial tries to understand patients’ satisfaction of receiving this form of intervention. Determining the clinical effectiveness and cost analysis of the intervention will help policy makers at the Ministry of Health as well as clinicians and other care-providers to improve their program planning.

## Abbreviations

SMI: Severe mental illness; ACT: Assertive community treatment; CMHT: Community mental health team; PANSS: Positive and negative symptoms scale; YMRS: Young mania rating scale; HDRS: Hamilton depression rating scale; CGI: Clinical global impression; GAF: Global assessment of functioning; WHOQOL-BREF: World health organization quality of life; CSQ-8: Client satisfaction questionnaire; GLM: General linear model; RCT: Randomized clinical trial.

## Competing interests

The authors declare that they have no competing interests.

## Authors’ contributions

All of the authors contributed to the design and development of the trial protocol. AH wrote the first draft of the paper that was finalized by VSh. All others commented and contributed to successive drafts. All authors read and approved the final manuscript.

## Pre-publication history

The pre-publication history for this paper can be accessed here:

http://www.biomedcentral.com/1471-244X/13/178/prepub
